# Assessment of quality and safety of meats from various animal species in the Shuchinsk-Burabay resort zone, Kazakhstan

**DOI:** 10.14202/vetworld.2021.1615-1621

**Published:** 2021-06-22

**Authors:** B. S. Maikanov, G. T. Ismagulova, L. T. Auteleyeva, Zh. O. Kemeshov, D. K. Zhanabayeva

**Affiliations:** Department of Veterinary Sanitation, Faculty of Veterinary Sciences & Animal Husbandry, S. Seifullin Kazakh Agrotechnical University, Astana, Republic of Kazakhstan

**Keywords:** amino acid rate, food safety, heavy metal salts, Shuchinsk-Burabay Resort Zone

## Abstract

**Aim:**

This study aimed to determine the food safety and protein adequacy of meats from various animals in the Shuchinsk-Burabay resort zone.

**Materials and Methods:**

Samples of meat were collected from markets “Zhomart” and “Kausar.” Two hundred and ninety-eight samples of meat were obtained: beef - 166, horse - 42, pork - 67, mutton - 8, and poultry - 15. From each carcass, part of the carcass, point samples were taken to form an average sample and conduct research. Analyses used inductively coupled plasma mass spectrometry and liquid chromatography. The determination of amino acid composition was carried out following the chromatographic method for determining the amino acid composition of meat proteins.

**Results:**

The quality of meats from the resort zone was lower than recommended due to the reduced content of essential amino acids, including valine, lysine, isoleucine, and phenylalanine. Concentrations of heavy metals and radionuclides did not exceed maximum permissible limits, and trace concentrations of arsenic, cadmium, and lead were detected in all meat samples, except beef. The latter meat showed increased concentrations of mercury.

**Conclusion:**

In terms of quality indicators, all types of meat met standards; however, pork protein displayed the most favorable amino acid composition, both for content of essential amino acids and the ratio of amounts of essential to non-essential amino acids. For content of heavy metals, poultry and pork meats were safest.

## Introduction

Food safety is always a major global political, economic, and life issue for governments and people. The safety of food sources continues to be a major challenge for production, consumption, and management [1]. Unsafe foods containing harmful bacteria, viruses, parasites, or chemicals cause more than 200 diseases ranging from diarrhea to cancer. An estimated 600 million people - almost one in 10 people worldwide fall ill after consumption of contaminated food each year and 420,000 dies. Thirty-three million years of healthy life are thus lost annually. Unsafe food causes a vicious cycle of disease and malnutrition, particularly affecting infants, young children, the elderly, and the sick [2].

The first global and regional study on foodborne disease (FBD) was initiated by the World Health Organization (WHO) in 2006. The following year, the WHO established the FBD Epidemiology Reference Group to guide the program. Nearly a decade later, initial estimates of the global burden of FBD were published. The combination of biological (28) and chemical (3) hazards was included in the study, and a significant global burden of disease was identified. For example, combined, these 31 hazards caused more than 600 million FBDs and 420,000 deaths in 2010. By themselves, these estimates are relevant; however, FBD disease burden estimates were comparable to estimates of the three major infectious diseases (i.e., HIV/AIDS, malaria, and tuberculosis: The “big three”). These appraisals were instrumental in drawing attention to a huge, underestimated, and global public health problem.

Biological hazards were identified in a WHO report as responsible for 97% of the total burden of disability-adjusted life years (DALYs), an indicator that provides an overall measure of the burden of disease. One DALY is a lost year of healthy life. The remaining 3% are due to just three chemical hazards (aflatoxin, cassava cyanide, and dioxin) [3]. In 2019, the World Bank approximated the economic costs of FBD [4]. Total loss of productivity associated with FBD in the LMIC is estimated at $95.2 billion per year, with middle-income countries accounting for $50.8 billion, and low-income countries for another $40.6 billion [4].

Sixteen resort zones are present in the Republic of Kazakhstan and are important for the development of tourism. A highly popular resort is the Shuchinsk-Burabay resort zone in the Akmola region [5]. Ecological recreation and socioeconomic development of the Shuchinsk-Burabay resort zone were studied by many scientists from Kazakhstan and neighboring countries (Begadilova and Golubeva). These researchers concluded that a resort zone is a favorite place for recreation for both residents from nearby administrative regions and tourists from CIS countries [6]. Shuchinsk-Burabay resort zone, a part of the “Burabay” State National Natural Park, includes elements of a nature reserve fund, mountains, lakes, and dendrological parks and arboretums.

Preliminary estimates indicate more than 170 sanatorium-resort and health-improving institutions, about 130 hotel complexes and tourist centers. These facilities combined possess about 12,000 beds in the resort zone. The most frequent products for vacationers, reflecting the food basket of Kazakhstanis, are meat, fish, and honey produced in the region. These products are widely used in sanatoriums, rest homes, and public catering centers. The resort zone’s advantageous location near the capital encourages an increase in the number of tourists from year to year. At present, 813,000 thousand visits per year are recorded. This figure is expected to reach 1.6 million visits by 2025. In 2019, the Burabay resort was the most popular among tourists from countries of the former CIS. Facilities for these tourists are growing every year. Anchor projects, such as the construction of the “AQBURA Resort” resort zone between lakes Bolshoye Chebachye and Tekekol, covering 233 hectares and worth 100 billion tenges, will increase the attractiveness of the Burabay resort for tourists [7].

The most important element of the resort area is the catering sector, which includes restaurants, bars, cafes, coffee shops, cafeterias, canteens, snack bars, fast food facilities, buffets, and cooking shops. Most catering services are included as basic services provided by resort health organizations. Catering organizations form an important part of the resort and recreational infrastructure, serving as a foundation for integrated function and development of the entire sanatorium and resort sector [8]. Food products of animal and plant origin, along with essential nutrients, might contain dangerous contaminants, such as cadmium, mercury, lead, and zinc, as well as macronutrients in excessive concentrations. Food safety is relevant for both developed and developing countries, especially in industrially advanced and ecologically unfavorable regions, as reported in studies of carcinogenic contaminants [9-11].

Contaminants are substances that are inadvertently intermixed with foods during production, processing, storage, and transportation. They may also mix with foodstuffs directly in the environment. The presence of such substances in food should be carefully monitored to avoid contamination that would compromise food quality or safety [12]. A growing interest in recent decades focused on the analysis of toxic metals in the aquatic environment, especially in measuring contaminant levels, radionuclide activity, and the effect of technogenic contaminants on the nutritional value of fish [13].

Scientists from different countries are reporting studies of heavy metals, radionuclides, and nutritional value in various matrices. For example, Parengam *et al*. [14] assessed Cd and Pb in rice sold in Thailand. In 2017, Abtahi *et al*. [15] analyzed As, Cr, Cd, Ni, and Pb in rice grown in Iran. The results showed an unacceptable hazard coefficient for consumers. Lanreiyanda and Adekunle analyzed As, Cd, Cu, Ni, Pb, and Zn in food products sold in Nigeria [16]. Ali and Al-Qahtani investigated Cd, Cu, Fe, Hg, Mn, Pb, and Zn concentrations in vegetables sold in Saudi Arabia [17]. Gupta *et al*. [18] investigated heavy metals in vegetables from irrigated wastewater. Concentrations of Cd, Cr, Pb, and Zn in vegetables exceeded MPCs of the national food safety standards of China. Finally, Rahmani *et al*. [19] analyzed 12 metals in canned tuna in Iran and conducted a health risk assessment.

Amino acid composition is determined in various animal and plant matrices. Mandume *et al*. [20] determined the nutritional value of meat from *Chaceon maritae*, an African marine crab. *C. maritae* protein proved to be of high quality and is a good source of essential amino acids. Essential amino acid content is higher than in reference amino acid sources and can supplement low-quality protein in other foods. Khazaei *et al*. [21] investigated the amino acid composition of lentil protein and found low levels of essential compared to non-essential amino acids. Chen *et al*. [22] studied the nutritional value of *Camellia sinensis* (black tea) and concluded that when the tea is fermented, and amino acid content is reduced. Kandyliari *et al*. [23] measured nutrient composition and nutritional value of fish. Traditional fish and fish by-products, as well as muscle tissue, contain essential amino acids, minerals, and vitamins. Studies of experimental toxicosis in mice administered heavy metals showed destructive changes in the gastrointestinal tract [24]. Ilyina and Semenova studied the migration of heavy metals along agricultural production chains and their effect on young cattle. Poisoning with these metals led to disruption of hematopoiesis and cell resistance [25]. In medical practice, when the territory of residence is contaminated with heavy metals, the proportion of spontaneous miscarriages, premature births, and neonatal pathology associated with miscarriage increases [26,27].

Overall, the literature on heavy metal contamination justifies safety concerns, especially in the resort areas of the Republic of Kazakhstan. Consuming unsafe and low-quality food is an important risk that tourists face during their travels. The Department of Food Safety, Zoonoses, and FBD (WHO) has developed a safety food guide for tourists [28].

This study aimed to determine the food safety and protein adequacy of meats from various animals in the Shuchinsk-Burabay resort zone.

## Materials and Methods

### Ethical approval

The research was approved by the ethical commission of the Faculty of Veterinary Medicine and Livestock Technology of the N. S. Seifullina KATU (extract from minutes No. 1 dated February 2, 2017).

### Study period and location

The study was conducted from March 2018 to May 2020. The study was carried out in the Shuchinsk-Burabay resort zone, Akmola region and in Nur-Sultan, the Republic of Kazakhstan. The studies were also carried out in the laboratory of food safety of “S. Seifullin Kazakh Agricultural and Technical University,” with further studies being conducted at the National Center for Expertise of the Committee for Quality Control and Safety of Goods and Services of the Ministry of Health of the Republic of Kazakhstan, in the testing laboratory of Nutritest LLP.

### Experimental design

The research study consisted of taking samples of meat of various animal species sold in the Shuchinsk-Burabay resort zone and determining protein adequacy and food safety.

#### Sampling

Samples of meat were collected from markets “Zhomart” and “Kausar.” Two hundred and ninety-eight samples of meat were obtained: Beef - 166, horse - 42, pork - 67, mutton - 8, and poultry - 15. Meat sampling followed State Standard 51477-99 [29].

#### Chemicals and reagents

All chemicals and reagents were of analytical quality and purchased from Sigma-Aldrich, Germany.

#### Determination of salts of heavy metals

Heavy metal concentrations in meats were assessed by inductively coupled plasma mass spectrometry (Agilent Technologies 7700, Japan). Analyses were performed at the Laboratory of Food Safety of RSE at RCEA “NRCV” Ministry of Agriculture of the Republic of Kazakhstan using standard operating procedure (SOP) “Determination of Trace Elements.” Arsenic, cadmium, mercury, and lead were measured after pressure mineralization by Agilent Technologies 7700 Series ICP-VS IC-SOP-M-E-002. This SOP was developed and tested based on British Standard BS EN 15763:2009 [30]. Measurement ranges were As: 0.06-21.5 mg/kg, Cd: 0.03-28.3 mg/kg, Hg: 0.04-–0.56 mg/kg, and Pb: 0.01-2.4 mg/kg.

Briefly, test solutions obtained by mineralization were sprayed and the aerosol fed into a high-frequency, inductively coupled argon plasma. High-temperature plasma was used to dry the aerosol and atomize and ionize the elements. Ions were extracted from the plasma using a set of cones (sampler and skimmer) and injected into the mass spectrometer. Ions were separated by mass/charge ratio. The detector determined values of pulsed or analog signals. The received signal was transformed into intensity on the value of m/z.

#### Determination of radionuclides

Sampling for radionuclides was carried out according to State Standard 32164 [31]. Analyses followed the Interstate Standard 32161-2013 [32] and State Standard RK 1623-2007 [33], using the spectrometric complex (Progress, The Russian Federation).

#### Determination of amino acid composition

The determination of amino acid composition was carried out following State Standard 34132-2017 [34]. Studies used a SHIMADZULC-20 Prominence liquid chromatography with a fluorometric and spectrophotometric detector (Shimadzu Corporation, Kyoto, Japan). Standard samples of amino acids (Sigma-Aldrich), acetonitrile (extra pure), isopropyl alcohol (extra pure), fluorescein isothiocyanate (FTIC; Sigma-Aldrich), phosphoric acid, and sodium acetate were used. In addition, measurement on a 25 cm×4.6 mm SUPELCO C18 used a 5 mm chromatographic column (Supelco Analytical, Bellefonte, PA, USA) with a guard column to protect the main column from impurities.

### Statistical analysis

Data were analyzed using Student’s t-test, and p<0.05 was considered statistically significant.

## Results and Discussion

### Amino acid

Amino acids are the basic units of proteins [35]. Complete meat proteins contain enough essential amino acids, and the quality of protein depends on its amino acid composition [36]. We identified changes in the qualitative and quantitative composition of amino acids in various meats (Table-1).

**Table-1:** Amino acids composition of meat protein in animals of different species.

#	Indicators	Pork	Beef	Poultry
Essential				
1	Valine	178.27±0.03	224.12±0.01	206.90±0.02
2	Isoleucine	448.67±0.04	525.24±0.004	520.42±0.03
3	Leucine	1304.79±0.004	1572.16±0.006	1438.60±0.003
4	Lysine	429.98±0.01	505.76±0.003	473.54±0.02
5	Methionine	473.29±0.02	629.83±0.02	543.34±0.01
6	Threonine	728.45±0.04	932.55±0.004	924.64±0.05
7	Tryptophan	339.91±0.004	307.27±0.004	468.95±0.004
8	Phenylalanine	438.66±0.07	525.55±0.01	487.12±0.03
	Total	6,609.36	5,222.48±0.004	5,418.11
Non-essential				
1	Arginine	1260.77±0.01	1469.79±0.02	1453.25±0.01
2	Alanine	1104.41±0.03	1197.77±0.04	1072.27±0.01
3	Tyrosine	1398.90±0.06	1684.71±0.03	1697.17±0.02
4	Cysteine	325.90±0.07	396.32±0.02	354.60±0.04
5	Aspartic acid	2221.11±0.02	2618.02±0.02	2610.46±0.05
6	Glutamic acid	3025.53±0.03	414.48±0.02	3559.22±0.05
7	Serine	820.33±0.04	990.85±0.04	937.17±0.04
8	Glycine	407.43±0.01	414.48±0.06	279.25±0.03
9	Proline	154.83±0.06	223.61±0.02	455.71±0,02
10	Histidine	856.80±0.05	865.54±0.02	777.86±0,02
	Total	9,738.62	14,105.5	13,315

The amino acid composition of pork protein was relatively low for essential amino acids, valine 21.5% of the norm; lysine, 34.7%; isoleucine, 63.4%; and phenylalanine 75.6%. The content of non-essential amino acids generally exceeded the norm by 1.3-2.6-fold, with exceptions of proline 154.83±0.06 mg/100 g (23.8% of the norm) and glycine 407.43±0.01 mg/100 g (58.6%). Similar results for beef protein were valine, 21.7% of the norm; lysine, 31.8%; isoleucine, 67.2%; and phenylalanine 66.1%. Non-essential amino acids exceeded the norm by 1.1-2.5-fold. Results for poultry meat were valine, 23.8% of the norm; lysine, 29.1%; phenylalanine 70.6%; and isoleucine, 71.3%. Non-essential amino acids exceeded the norm by 1.1-2.7-fold.

Proteins and amino acids do not accumulate in the body, and excesses of some amino acids identified above will not adversely affect human health. When eating foods containing an increased content of amino acids in their composition, excess amino acids will either be excreted or metabolized. The nitrogen in amino acids is converted into urea and creatinine and excreted by the kidneys. The carbon skeleton is used for the biosynthesis of glucose and fatty acids, and side chains are oxidized to carbon dioxide and water with the formation of ATP. In contrast, deficiency in a single essential amino acid may lead to growth retardation, weight loss, impaired energy metabolism, dysfunction in human systems, and organs [37,38].

Our data on valine content in the meat from warm-blooded animals correlate with data on fish proteins. These proteins are deficient in valine (60-62%), isoleucine (59-79%), tryptophan (42-63%), and phenylalanine (76-81%), although they contain more lysine and methionine [37]. Meat proteins of various animal species are not adequate in terms of valine and isoleucine content; this deficiency can be compensated by consumption of protein with increased content of valine and isoleucine. The concentration of the remaining essential amino acids exceeded the norm by 1.1–3-fold. Excess amino acid content is not critical, since heat treatment (cooking) of meat leads to a decrease in the amino acid content [39].

The ratio of the sum of essential to non-essential amino acids in protein of pork - 0.68, beef - 0.37, and poultry meat - 0.40 was higher than the norm (Table-2). Meat samples also differed from the norm in the amount of amino acids: Pork by 13%; beef, 4%; and poultry meat by 2.6%. Nutritional value using a standard based on an ideal protein indicates that limiting amino acids in various meat samples are valine (22.3-28% of the norm) and isoleucine (70.1-82.1%) (Table-3). Under the influence of technogenic and biogenic factors, the nutritional value of meat protein increased for isoleucine, leucine, phenylalanine, and valine, which correlates with our results [40,41].

**Table-2:** Amino acid indicators of meat protein.

Indicators	Pork	Norm	Beef	Norm	Poultry	Norm
Ratio of the sum of essential to non-essential amino acids	0.67	0.67	0.37	0.63	0.40	0.60
Total sum of amino acids	16,347.98	16,927	19,327.98	18,429	18,734.01	18,230

**Table-3:** Amino acid rate of meat protein samples.

Indicator	Pork	Beef	Poultry
Valine	22.3	28	25.9
Isoleucine	70.1	82.1	81.3
Leucine	116.5	140.4	128.4
Methionine + cystine	142.7	230.2	103.5
Threonine	113.8	145.7	144.4
Tryptophan	212.4	192	293
Phenylalanine + tyrosine	191.4	230.2	227.5

### Heavy metals and metalloids

Heavy metals were assessed in different seasons and horse meat and mutton were also investigated. Horse meat was consumed in autumn and winter, while mutton in summer and spring. Cadmium 0.01±0.0002 mg/kg, mercury 0.02±0.0001 mg/kg, and lead 0.1±0.0003 mg/kg were detected in horse meat samples. In beef samples, mercury concentration was 0.01±0.0002 mg/kg and lead –0.1±0.00001 mg/kg. Arsenic concentration in mutton was 0.01±0.0001 mg/kg, mercury 0.03±0.0002 mg/kg, and lead 0.1±0.00001 mg/kg (Table-4). Heavy metals were detected in poultry meat. Mercury content is at the upper limit of the norm in beef and mutton samples. Prolonged consumption of meat containing heavy metals may lead to disease. For instance, the accumulation of cadmium in the body contributes to the development of brain cancer [42]. We found cadmium in horse meat samples.

**Table-4:** Contents of heavy metals salts in various species meat samples, mg/kg.

Item	As (arsenic)	Cd (cadmium)	Hg (mercury)	Pb (lead)
Maximum permissible concentration	0.1	0.05	0.03	0.5
Horse meat (n=42)	<0	0.01±0.0002	0.02±0.0001	0.1±0.0003
Pork (n=67)	<0	<0	<0	<0
Beef (n=166)	<0	<0	0.03±0.0263	<0
Mutton (n=8)	0.01±0.0001	0	0.03±0.0002	0.1±0.00001
Poultry (n=15)	<0	<0	<0	<0

Bold values indicate that the concentration of mercury in products has reached the MPC

Chronic exposure to heavy metals may result in the accumulation of toxic concentrations in the body. Long-term exposure to cadmium may cause “itai-itai” (oh-oh) disease identified in a Japanese population. The name of the disease comes from pain in the back and legs that accompany osteomalacia (decalcification) and fragility of bones. Chronic cadmium poisoning also damages the liver and kidneys, resulting in severe renal dysfunction. An excess of cadmium disrupts the metabolism of metals, especially iron and calcium, disrupts the action of zinc, and affects metal enzymes, blocks sulfhydryl groups, and disrupts DNA synthesis. Cadmium disrupts the two-stage oxidation process by replacing other metals in metal-flavoprotein complexes, where iron and molybdenum play a dominant role.

Mercury was found in samples of beef, mutton, and horse meat. Mercury is toxic in any form. In the body, methyl mercury rapidly enters erythrocytes, liver, and kidneys. It also accumulates in the brain, causing serious irreversible disorders of the central nervous system. This ultimately leads to general and cerebral palsy, deformation of the limbs, especially fingers, difficult swallowing, convulsions, and death. Mercury blocks the activity of several important enzymes, in particular carbonic anhydrase, carboxypeptidase, and alkaline phosphatase. Hg replaces cobalt in corrinoids, antagonizing the metabolic reactions associated with Vitamin B12. Disruption of DNA biosynthesis due to Vitamin B12 deficiency is a cause of megaloblastic anemia, including the most common form, pernicious anemia, which leads to degenerative changes in the nervous system.

Lead was found in horse meat and mutton samples. At present, the largest source of lead pollution in the environment is associated with exhaust from gasoline engines due to the addition of tetraethyl lead to gasoline to increase the octane rating. Lead interferes with one stage of heme biosynthesis and is a notable neurotoxin. Chronic lead poisoning gradually leads to impaired renal function, anemia, and nervous system disruption, including increased aggressiveness. The toxicity of lead increases with calcium and iron deficiency. Lead blocks SH-groups of proteins, forms complexes with phosphate groups of ribose, especially cytidine, and thereby rapidly destroys RNA, and inhibits enzymes, notably, carboxypeptidase.

Arsenic, a potent and dangerous poison, was found in mutton. High concentrations of arsenic are observed in the stomach, intestines, liver, kidneys, and pancreas after oral exposure. Chronic exposure results in the accumulation of as in skin, hair, and nails. As also inhibits various enzymes and thus disrupts metabolism. Axons are the first to suffer, leading to peripheral neuropathy and paralysis of the limbs. Arsenic is considered carcinogenic to humans [43]. Heavy metals in ecosystems come, ultimately, from parent rock. The release of these elements often occurs because of human activity. Soil pollution around industrial centers occurs mainly through releases from industrial enterprises and environmental transport. Anthropogenic sources of heavy metals in the biosphere are numerous, such as metalworking wastes, industrial emissions, fossil fuel combustion products, exhaust gases and liquids from automobiles, and agricultural chemicals. Natural sources of heavy metals also contribute through the decomposition of mineral rocks during soil formation, release into the atmosphere during volcanic eruptions, natural fires, and weathering of rocks. Cd, Pb, Zn, Hg, and Cu are industrial products and important environmental pollutants [44,45].

### Radionuclides

Cesium-137 was recorded as 13.47±2.9633 Bq/kg in beef samples, 13.47±2.9633 in horse meat, 11.3±1.9712 in pork, and 6.7±2.7056 in poultry (Table-5). The activity of cesium-137 did not exceed the maximum permissible level in any meat sample. Meat from the resort zone does not present a health hazard, likely because no heavy industry or mining operations exist in the Shuchinsk-Burabay resort zone.

**Table-5:** Average values of cesium-137 contents in meat samples.

Meat samples	Research results, Bq/kg	Permissible level, Bq/kg
Beef (n=166)	13.47±2.9633	200
Horse meat (n=42)	14.37±7.2972	200
Pork (n=67)	11.3±1.9712	200
Mutton (n=8)	4±0.0289	200
Poultry (n=15)	6.7±2.7056	200

## Conclusion

Thus, in terms of quality indicators, all types of studied meat corresponded to the standards, however, the protein of pork meat had the most adequate composition, both in terms of the content of essential amino acids and the ratio of the amount of essential amino acids to non-essential ones. The least adequate was beef meat protein, while valine and isoleucine were the limiting amino acids in beef and poultry. Samples of poultry and pork were the safest in terms of the content of heavy metal salts. In beef and mutton, a threshold for mercury contents has been set (0.03 mg/kg).

## Authors’ Contributions

BSM: Conception and design, analysis and interpretation of data, and drafting of the manuscript. GTI: Acquisition of data and drafting of the manuscript. LTA: Conception and design, analysis and interpretation of data, and drafting of the manuscript. ZOK and DKZ: Revised the manuscript. All authors read and approved the final manuscript.
